# Commentary: Efficacy and Safety of Chinese Herbal Medicine on Ovarian Cancer After Reduction Surgery and Adjuvant Chemotherapy: A Systematic Review and Meta-Analysis

**DOI:** 10.3389/fonc.2020.565812

**Published:** 2020-10-28

**Authors:** Mingxing Li, Miaofa Ying, Rui Zhao

**Affiliations:** Department of Pharmacy, Sir Run Run Shaw Hospital, School of Medicine, Zhejiang University, Hangzhou, China

**Keywords:** Chinese herbal medicine, ovarian cancer, meta-analysis, comment analysis, efficacy and safety

In a previous issue of *Frontiers in Oncology* published in August 2019, we read with great interest the article by Wang et al. (1) entitled “Efficacy and Safety of Chinese Herbal Medicine on Ovarian Cancer After Reduction Surgery and Adjuvant Chemotherapy: A Systematic Review and Meta-Analysis.” The authors performed a meta-analysis to assess the efficacy and safety of Chinese herbal medicine (CHM) in the treatment of ovarian cancer after reduction surgery and adjuvant chemotherapy. The study is of great value and provides a proper regimen for the treatment of ovarian cancer in the future. However, there are still some flaws that we would like to discuss with the authors.

First, only two electronic databases (CNKI and PubMed) were searched by the authors, and 18 studies from China were included. According to the Preferred Reporting Items for Systematic Reviews and Meta-Analysis statement criteria (2), the proper search strategies and adequate studies are essential when reporting a meta-analysis. Therefore, we suggested that more electronic databases should be systematically searched, including the Wanfang database, Cochrane Library, Embase, and Medline, and detailed search protocols should be provided by the authors.

Second, all the included studies came from Chinese journals in this study, and the details of quality assessment of all the studies were not provided. In order to ensure the accuracy of the results, all studies should be scored according to the Jadad Scale (Table 1). In addition, according to the Cochrane collaboration's tool (3), the methodological quality and the risk of bias of each study should be evaluated by two investigators. However, the authors did not conduct the evaluation in their meta-analysis. In our opinion, the methodological quality and the risk of bias should be performed so as to eliminate the low-quality studies.

**Table 1:** Jadad scale.

**Items**	**Scores (0–7)**		
	**0**	**1**	**2**
Randomization	Not randomized or inappropriate method of randomization	The study was described randomized	The method of randomization was described, and it was appropriate
Double blinding	No blind or inappropriate method of blinding	The study was described double blind	The method of double blinding was described and it was appropriate
Concealment of allocation	Not describe the method of allocation concealment	The study was described allocation concealment	The method of allocation concealment was described, and it was appropriate
Withdrawals and dropouts	Not describe the follow-up	The study was described withdrawals and dropouts	

Third, Q test and *I*^2^ test were used to assess heterogeneity in their meta-analysis. According to the Cochrane Reviewers' Handbook, the fixed effect model was used when *I*^2^ value was less than 50%, otherwise the random effect model would be applied. In their article, subgroup analyses were used to evaluate the urinary system symptoms, and the results are shown in Figure 3C. Although the values of *I*^2^ were no more than 50%, the random effect models were still used by the authors in Figures 3C,D,F. We hope to get the authors' viewpoint on this issue. In addition, the results of peripheral neuropathy are showed in Figure 3E, and not in Figure 3F, a mislabeling. Meanwhile, we think that sensitivity analysis is still necessary to ensure the accuracy of the results.

Fourth, even though the risk of bias was assessed using the Cochrane risk of bias tool (4), the publication bias was not mentioned in their meta-analysis. As far as we know, the funnel plots should be used to evaluate the publication bias.

All in all, we are grateful to the authors for their contribution; they summarized the safety and efficacy of CHM in the treatment of ovarian cancer after reduction surgery and adjuvant chemotherapy. However, rigorous preclinical high-quality RCTs involving CHM in the treatment of ovarian cancer is still needed to reach rational conclusions.

## Author Contributions

All authors listed have made a substantial, direct and intellectual contribution to the work, and approved it for publication.

## Conflict of Interest

The authors declare that the research was conducted in the absence of any commercial or financial relationships that could be construed as a potential conflict of interest.
